# Exploring Factors Associated With Family Caregivers’ Preparedness to Care for an Older Family Member Together With Home Care Nurses: An Analysis in a Swiss Urban Area

**DOI:** 10.1177/21501319221103961

**Published:** 2022-06-07

**Authors:** Irène Ris, Thomas Volken, Wilfried Schnepp, Romy Mahrer-Imhof

**Affiliations:** 1 ZHAW Zürcher Hochschule für Angewandte Wissenschaften, Winterthur Schweiz, Switzerland; 2 Universität Witten/Herdecke, Fakultät für Gesundheit, Witten, Deutschland; 3 Nursing Science & Care Ltd, Winterthur, Schweiz

**Keywords:** long term care, community health, geriatrics, quality improvement, health outcomes

## Abstract

**Introduction:**

Home-dwelling older people with chronic diseases often need the support of informal and formal caregivers in order to continue living at home. Family members, however, need to be willing and prepared for caregiving together with home care nurses.

**Objectives:**

The purpose of this study was to explore factors associated with family caregivers’ preparedness to care for older home-dwelling adults who also receive home care nursing services.

**Methods:**

For this cross-sectional correlational study, a structured questionnaire was sent to family caregivers of adults aged 65 years or older receiving services from a community care agency. A total of 243 participants returned the questionnaire, of which 199 could be analyzed.

**Results:**

The stepwise backward regression model explained 29.1% of the variance of family caregivers’ preparedness. Mutuality was the most strongly associated factor with family caregivers’ preparedness whereas professional involvement of family caregiver in care process was important as well. Care intensity showed no significant impact.

**Conclusion:**

Nurses should support the whole family emotionally, and appreciate, admire, reinforce, and respect the caregivers’ situation. Home care nurses need to invest in helping families to find solutions, to strengthen their relationships between family members and the older person dwelling at home.

## Introduction

With an aging population, societies are faced with increased numbers of older adults needing various forms of assistance (Organisation for Economic Co-operation and Development [OECD]).^1^ Elderly people strive for independence and wish to dwell at home as long as possible, even when in need of support.^2^ Health policy also favors “aging in place” and prioritizes community care over residential care.^3^ Higher life expectancy and an increase of chronic diseases in old age will lead to higher numbers of older adults dwelling at home requiring support.

This vulnerable population often receives both formal care from home care nursing services as well as informal care from their next of kin. It is known that family caregivers play a pivotal role so that older adults with care needs can age in place.^4-8^ A collaboration between the care receiver, family caregivers and nurses must be established, and a care triad formed. Nursing support in the care triad is seen as a resource for the family unit, by lowering stress in family caregivers and increasing satisfaction with the caregiving situation.^9,10^ Previous research showed that involving family caregivers as a co-experts in the care team enabled family caregivers to gain self-confidence to maintain family routines and intimacy with the care receiver, as well as to sustain the caregiving over the long term.^11-14^ Therefore, nurses need accurate knowledge of the family system to support family caregivers within the care triad.^15,16^

Family caregivers’ perception about their readiness to adopt and maintain the caregiver role is referred in the literature as preparedness,^17^ and has been studied in patients with heart failure,^18^ patients receiving palliative care or cancer treatment,^19,20^ and patients living with dementia or Alzheimer’s disease.^21,22^ In these studies, high preparedness scores have been identified as a predictor for positive family caregiver outcomes such as lower levels of anxiety and depression, less burden or higher states of hope, and quality of life.^18-20^ Additionally, family caregivers’ preparedness has been positively associated with care receivers’ outcomes including reduced pain levels, increased functional status, and improved mental health.^23^ But the perception of family caregivers’ preparedness can also be understood as a process of adaption. According to the family resilience theory, the process of preparedness depends on the family adjustment to the precipitating events which lead to positive or negative emotions within the care triad.^6,24-28^

The Family Adjustment and Adaptation Response (FAAR) Model developed by McCubbin and Patterson^29^—a family resilience theory—highlights that families are constantly engaged in dealing with demands such stressors, strains, and daily hassles, balanced against the family’s ability to cope, to assign meaning, and to address context factors.^15,25^ Good adaptation to coping with the caregiving demands leads to smooth functioning within the family. These families may be labeled as resilient or in other words, well prepared for their caregiving challenges and experiences.^15,16,30,31^

According to the literature, family caregivers’ characteristics influenced the caregiving experience. For example, older family caregivers experienced caregiving more positively than younger caregivers, but showed higher levels of depression, memory loss, and anxiety.^32-34^ Women reported more negative experiences than men,^28,31^ but felt significantly better prepared for caregiving.^20^ Individuals with a higher education had more self-confidence in the care they provided.^12,15,34^ Incidentally, family caregivers with lower education received more support from community nurses.^35^ Studies which investigated whether the care receivers’ characteristics have an effect on caregivers’ preparedness had shown inconclusive results. Whereas the amount of caregiving tasks needed to support activities of daily living predicted the degree of caregiver burden in one study,^26^ other studies showed that the severity of care receiver’s disability and support needs were not significantly related to caregiver burden.^12^ A moderate positive correlation was found between care hours delivered by family caregiver per week and the burden experienced.^6,24,26-28^

Good relationships within family seem to be an important factor for engagement in caregiving and overall preparedness. Mutuality, a term for the relationship quality between the family caregiver and care receiver, has been widely reported to affect caregiver outcomes.^36^ For instance, a better relationship with the person in need of care increased satisfaction and the caring expertise of the family caregiver.^37^ Mutuality within the family and the readiness for caregiving are positively associated,^19,21,38-41^ as are living arrangements. “Living together” in a shared home, compared with “living apart,” required less coordination of time, activities and resources and had a positive impact on family satisfaction.^32,42^

Finally, nursing support in the care triad has been shown to lower stress in family caregivers and to increase satisfaction with the caregiver situation.^9,10^ Involving family caregivers may enable families to maintain and sustain the caring situation,^11-14^ but little is known which family caregivers would benefit most from involvement and support by nurses from community services to strengthen the care triad. Therefore, the study aimed to explore factors associated with family caregivers’ preparedness from the perspective of the family caregiver as a part of the caregiver triad.

## Methods

This cross-sectional and correlational study used structured questionnaires to collect data. Derived from the Family Adjustment and Adaptation Response (FAAR) model, 16 possible factors associated with preparedness in the care triad have been explored. The factors may represent either demands, such as care intensity and duration of care, or capabilities, such as high mutuality and involvement of care.

### Recruitment and Sample

A collaborator working at a community care agency in a major city in the German-speaking part of Switzerland identified potential family caregivers through file review. Family caregivers were defined as people that provide unpaid care to a home-dwelling person and considered themselves to be a family member based on emotional, biological, or economic ties.^43^ From a total of 1257 care receivers aged 65 or older, 1672 family caregivers were invited to participate in the study.

Care receivers were first informed in writing about the study and were given an opportunity to decline having the study materials sent out to their family caregivers. If the care receiver did not mail in non-approval (ie, opted out), the study materials were sent to the family caregivers, with one follow-up reminder 1 month after the initial notification.

Both the family caregiver and the care receiver were informed that the entire participation process was voluntary and that the data were anonymized. The ethics committee of the canton Zürich approved the study (KEK-ZH-BASEC-Nr. Req.-2016-00288).

### Data Collection

Data collection in a convenient sample was conducted in 2017 by sending study information, informed consent, the questionnaires and a return envelope to the family caregivers. The structured questionnaire comprised a total of 70 questions, which were divided into 5 sections: (1) characteristics of the family caregiver and care receiver; (2) care demands; (3) relationship within the family; (4) preparedness; and (5) involvement in care. An estimated 40 min was needed to complete the questionnaire.

### Variables and Instruments

#### Characteristics

The characteristics of the family caregiver and care receiver included age in years, gender, and marital status (married or partnered, single, divorced, or widowed). Additionally, family caregivers’ education level (mandatory elementary education and secondary school or high school and higher education) was assessed.

#### Relationship within the family

Relationship status and living arrangement of family caregiver and care receiver were determined, and the quality of the relationship assessed.

Relationship status was defined as existing between the care receiver and members of their immediate family (partner, sibling, own child) or extended relations such as friends, neighbors, or other individuals.

The German version of Mutuality Scale of the Family Care Inventory [FCI] was used to investigate relationship quality.^30,38^ The FCI is a 15-item scale, which reflects the interactive nature of relationship quality between the family caregiver and care receiver, and includes dimensions of reciprocity, love, shared pleasurable activities, and shared values.^38^ Sample items are: “How close do you feel to him or her?” “How much do the two of you laugh together?” or “How much emotional support does he or she give you?” Family caregivers responded to items using a Likert-scale from 0 (not at all) to 4 (a great deal) and a score is calculated as the mean of all items.^39,40^ Cronbach’s alpha values were between .91 and .95.^36,44-46^

The living arrangement was assessed with a dichotomous variable (living together with care receiver or living apart).

#### Intensity of care demands

To measure the degree of dependence of the care receiver we used the multidimensional Functional Assessment Questionnaire “Older Americans Resources and Services” (OARS) which assesses activities of daily living such as bathing, dressing, toileting, transfer, and eating (5 items) and instrumental activities of daily living such as cooking, shopping, cleaning, managing money, administrating medications, and using the telephone (7 items).^47^ The score ranges between 0 and 28, a lower score indicating higher dependency. The Cronbach’s alpha was reported from .86 to .90.^47,48^

The duration of family caregiving was assessed by months of caregiving and hours per week.

Family caregivers were asked to indicate the care hours delivered by the community care service and to differentiate between nursing services and domestic support (eg, housecleaning).

#### Professional involvement of family caregivers in care process

Family caregivers’ involvement in care was assessed with the subscale of perceived social support from the Family Functioning, Health and Social Support [FAFHES] questionnaire.^49^ The questionnaire assesses the perceived support and recognition toward families provided by nurses.

The subscale contains 3 domains: first the “affective” or emotional support that measures the perceived appreciation, admiration, sense of security, respect or love; second “affirmation” that describes reinforcement, feedback and help the individuals to find a solution; and third “concrete aid,” meaning spending time, helping someone with tangible support, for example, preparing a meal, organizing services, or providing financial means.^50,51^ The questionnaire has been used across settings and populations, including families with children and older people living at home. It has been translated into different languages.^9,51-56^ The internal consistency coefficients showed Cronbach’s alphas > .90.^9,56^

#### Dependent variable preparedness

Preparedness was assessed with the Preparedness-Scale by Schumacher et al.^30^ The scale consists of 8 items to evaluate how prepared family members feel for caregiving tasks, such as providing physical and emotional care or coordinating the organization of services. Items include, “How well prepared do you think you are to take care of your family member’s emotional needs?” and “How well prepared do you think you are to get the help and information you need from the health care system?” A 5-point Likert scale is used to rate each item (0 = not at all prepared; 4 = very well prepared), the index represents an average value over all items of the scale.^38^ Cronbach’s alphas of this widely used questionnaire have been reported between .87 and .92.^44,45,57^

### Data Analysis

Data were entered into the IBM SPSS Statistical Programme version 26. Multiple imputation methods (estimation maximization) helped to compensate for missing data when 3 or fewer items were missing, by imposing a probability model on the completed data.^58^ If more than 20% of the data (ie, 4 or more items) of social support scale, preparedness, and mutuality were missing, the cases were excluded. In a first step, the characteristics of study participants were assessed using descriptive statistics (mean and standard deviation or percentages, Table 1). To explore associations among the dependent variable, preparedness, and the candidate covariates, we used multivariable linear regression models with backwards elimination with a probability level>.1. The backwards elimination approach was motivated by Occam’s razor, that is, the idea that if several explanations are compatible with a set of data, the simplest—including the fewest possible parameters—should be chosen. Mean-centered predictor variables were used throughout the analysis for all continuous predictors to eliminate multicollinearity when interaction terms entered the regression equations.^59,60^

**Table 1.** Characteristics of Participants and Variables (n = 199).

	*M*	SD	n	%
Characteristics family caregiver FC				
FC age in years	62.3	11.30		
Gender				
Male			75	37.7
Female			124	62.3
Marital status FC				
Married or partner			140	70.4
Single, divorced or widowed			59	29.6
Education FC				
High school, higher education			95	47.7
Mandatory education			104	52.3
Characteristics care receiver CR				
CR age in years	84.6	9.03		
Gender				
Male			57	28.6
Female			142	71.4
Marital status CR				
Married or partner			52	26.1
Single, divorced or widowed			147	73.9
Relationship within family				
Nuclear family			156	78.4
Friend or other			43	21.6
Living situation FC				
Together with CR			47	23.6
Apart from CR			152	76.4
Mutuality	2.9	0.87		
Intensity of care demands				
OARS score	16.0	6.38		
ADL	8.8	3.14		
IADL	7.2	3.68		
Care hours per week FC	18.4	32.33		
CR duration of care (in months)	62.4	61.91		
Professional family support				
Involvement	55.5	23.90		
Nursing care hours	4.8	3.87		
Nursing service				
Domestic support			39	19.6
Nursing and domestic support			160	80.4
Dependent variable				
Preparedness	2.5	0.77		

Abbreviations: FC, family caregiver; CR, care receiver.

For all linear regression models, we visually checked whether independent variables were related linearly to the outcome, checked homoscedasticity using the Breusch-Pagan test, and assessed multicollinearity using the variance inflation factor (VIF) in the multivariable models. We reported unstandardized and standardized regression coefficients with corresponding standard errors (SE), *t*-values, and *P*-values.

Significant determinants will be interpreted according the FAAR Model, where positive correlation will be linked to capability while negative correlation corresponds to demands. Statistical significance was established at *P* < .05.

## Results

A total of 243 questionnaires were returned, but due to missing data, 44 surveys had to be excluded. In the end, a total of 199 questionnaires could be analyzed.

The mean age of the care receivers was 84.6 years (±9 SD) with a range from 65 to 98. Two-thirds of the care receivers were women (71.4%; 142), with the majority single, divorced or widowed (78.4%; 147). The care dependency measured with the OARS was 16 (±6.38 SD).

The mean age of the family caregivers was 62.3 years (±11 SD, Mdn = 60, IQR = 55–70), with a wide range between 31 and 90. Of 199 family caregivers two-third (62.3%; 124) were women, more than two-third (70.4%; 140) were married or lived in partnership. Almost half of the family caregivers (47.7%; 95) had more than the mandatory level of elementary education.

Family caregivers were mainly partners, siblings, or adult children (78.4%; 156). More than three-quarters (76.4%; 152) of family caregiver and care receiver were living apart. The average on the mutuality scale, measuring relationship quality between the family caregiver and care receiver, was 2.9 (±0.87 SD).

The family caregivers provided an average of 18 h of care per week (±32.33 SD, Mdn = 6, IQR = 3–15), ranging from 1 to 168 h. The duration of care was on average of 62.4 months (around 5 years).

The perceived involvement in care from professionals had an average score of 57.6 (±23.9 SD), with a range from not involved (0) to very involved (95). The home care agency provided home care nursing to at least four-fifths (80.4%; 160) and household help to one-fifth (19.6%; 39) of the care receivers. The time professionals spent for both types of services ranged from 45 min to 23 h per week, with an average of 4.8 h (±3.87).

Preparedness, the dependent variable, had an average score of 2.5 (±0.768 SD).

Overall, the model explained 29.1% of the variance of preparedness. Five variables were significantly associated with preparedness: being married as care receiver, family caregiver’s gender (borderline significance with *P* = .051), the number of care hours delivered by the family caregiver per week, being a friend that served as a family caregiver, perceived mutuality with the care receiver, and involvement in care. Family caregiver’s gender was of borderline statistical significance^61^ with *P* = .051 (see Table 2). Considering standardized beta coefficients, the independent variables mutuality and not being a member of the immediate family had the highest values, indicating that in terms of effect size, they had more importance relative to other factors in the model.

**Table 2.** Regression Coefficients of Preparedness (n = 199).

Variables	*B*	SE *B*	β	*t*	*P*
(Constants)	2.658	0.081		32.998	.000
Marital status CR	0.234	0.117	.134	1.996	.047
FC gender	−0.189	0.096	−.119	−1.963	.051
Care hours per week FC	0.003	0.002	.147	2.320	.021
Relationship within family	0.346	0.119	.186	2.896	.004
Involvement	0.006	0.002	.185	2.808	.005
Mutuality	0.339	0.056	.384	6.068	.000
Model adjusted *R*^2^	0.291				.000

Abbreviations: CR, care receiver; FC, family caregiver.

*R*^2^ = 0.313.

## Discussion

This cross-sectional correlational study examined the preparedness of family caregivers and its associated factors. Five factors including relationship quality, perceived involvement of family caregivers in the care triad, relationship status, the number of care hours delivered per week by a family caregiver, and marital status were significantly associated with preparedness and explained 29.1% of the variance.

Generally, family caregivers’ preparedness was fair, with the average score of 2.9 and was similar to other studies showing scores between 2.1 and 2.8.^18,22,30,57^ The response rate with 14.5% was rather low but comparable to other Swiss studies with similar populations (7.7%-21.7%).^62,63^ Our sample was comparable to samples in other studies regarding gender, age and relationship status,^1,2^ but differed by showing lower levels of mutuality (2.9 vs 3.4).^57^ This result might be due to the 43 family caregivers who labeled themselves as “friend or other”. In the free text field of the questionnaire, 34 persons of those 43 “friends” were voluntary representatives. Therefore, we assume that they might perceive less mutual emotions and closeness with the care receiver. Additionally, studies with a similar population suggested that mutuality might decline over time and length of caregiving.^36,57,64^ This might also be true for our sample, which had an average duration of caregiving lasting 5 years. Compared with other studies, the care receivers had higher OARS scores, indicating more dependency. The number of care hours provided by family members (18 h per week) indicate intensive caregiving duties, as caregiving above 11 h per week has been defined as intense.^2^

The most important factor in our model was mutuality, one dimension of relationship within family. High perceived mutuality was associated with high preparedness scores. This finding is in accordance with previous investigations in which mutuality correlated positively with preparedness.^21,38,64^ These results underline that home care nurses should involve the family as a unit, in order to function as a team throughout the care trajectory potentially deepening the family relationship.^64^ The relationship dimension to be married as family care receiver and to be a friend showed to be relevant for preparedness as well. Being married can be interpreted according the FAAR Model as a meaning to accept the caregiver role. Family caregivers of married care receivers felt better prepared than those of care receivers who were single, divorced, or widowed, even when the care receiver was not married to the caregiver. Nevertheless, the effect of a normative obligation of marriage may provide sufficient explanation, as mentioned above and in a previous study.^12,65^ The living arrangement as a further dimension of relationship did not influence preparedness in our sample.

The perception of being involved as family caregiver was shown to be an important factor in our model as well. As in previous studies, we found that being supported and recognized by professionals in the care triad had a significant impact on family caregivers’ preparedness. This finding underlines the crucial role of nurses in collaborating with family caregivers to ensure optimal care for care receivers. Earlier studies have shown that family caregivers perceived that preparedness increased the health and self-care ability of older persons with chronic conditions.^23^ Although we did not assess how the care receiver perceived involvement, it is very likely that the care receivers have benefited as well. However, further studies are needed to clarify this issue.

Factors in our model that can be assigned to the care intensity dimensions or as a demand according the FAAR model may not necessarily influence preparedness. The intensity of care demand measured with the OARS was not a significant factor. Therefore, the severity of disability which affects basic daily activities of the care receivers, may not influence preparedness. The finding is in accordance with the literature, which did not describe dependency as a major factor,^12,26,66^ whereas the numbers of care hours per week was significantly related to higher preparedness scores. Although this result might be surprising at first sight, the literature shows that family caregivers may become more aware of their caregiver role and develop expertise when spending more time per week with the care receiver.^2,23^ While the hours of care provided by family caregivers showed an effect on preparedness, the length of time or duration caregiving does not appear to be an important issue for family caregivers’ preparedness. This might be due to the cross-sectional design of our study, which does not capture the adjustment process over time that has been described in the literature. The care required by the older adults requires adjustment over time due to changing illness.

Finally, characteristics of the family caregivers and care receivers may be interpreted according the FAAR model as context factors, which effect preparedness.^36^ We did not find associations among age or education level of family caregivers, nor the age of the care receiver with preparedness. In contrast, Petruzzo et al^18^ findings showed an inverse relationship between age of caregivers of heart failure patients and preparedness. Moreover, previous studies found associations of level of education with preparedness.^23^ Only the finding that women were better prepared than men is consistent with previous studies.^20^ According to the FAAR Model, adjustments between care demands and capabilities may differ by gender^67^ and a perceived female obligation to care may have had an effect on feeling better prepared.^68^ Therefore, nurses should be aware of gender vulnerabilities which exist in the day-to-day work with families.

### Limitations

In our explorative regression model, we included several parameters based on relevant literature. While we consider our model to reflect a snapshot of the care triad, we may have missed other important factors that may be relevant to the care triad. The latter may potentially have induced omitted-variable bias. Moreover, the limits of a cross-sectional study design need to be considered. Using a family caregiver self-report questionnaire may have resulted in an over-or underestimation of determinants. For example, the item “caregiving hours” provided by family caregivers and home care nurses may not be enough reliable and valid, considering the wide range of the variable. Moreover, the use of self-report measures could have increased the risk of social desirability response bias affecting the results. Furthermore, the interpretation of results within the context of the FAAR Model should be made with caution, as we interpreted that mutuality in combination with involvement may balance the care demands and yet in the end effect, care demands were not significant in the model. In addition, results around gender, marital status and not being a member of the immediate family are difficult to interpret with the theoretical approach used. Finally, we used standardized regression coefficients to assess the relative importance of effects, but any effect reflected by the standardized coefficients may be due to confounding with the particularities of our sample, such as the variability and distribution of specific variables.^69,70^

## Conclusion

The family caregivers from this study were fairly prepared for providing care to care receivers. Mutuality and professional involvement were positively associated with preparedness whereas intensity and demands were not relevant. To increase quality care, nurses play a pivotal role in supporting family relationship regardless of care intensity. Additionally, the positive association of not being a member of the immediate family with preparedness indicates that nurses need to tailor their intervention to the caregiving arrangement and think beyond supporting only members of the immediate family. They also can help to overcome gender stereotypes. For example, male family caregivers—single, divorced, or widowed—have been shown to be less prepared and may need special attention in clinical practice.

Our study also showed the need for further research. Exploring more factors from the perspective of nurses or care receivers and its influence on family caregivers’ preparedness need to be conducted to develop a stringent theoretical framework. Longitudinal studies are needed to investigate changes over time. For example, the impact of preparedness and involvement by nurses on caregivers’ health or on length of home dwelling should be investigated in future.
